# Development of an anti-inflammatory diet for first-episode psychosis (FEP): a feasibility study protocol

**DOI:** 10.3389/fnut.2024.1397544

**Published:** 2024-07-26

**Authors:** Leda Kennedy, Tiffany Holt, Anna Hunter, Shahrokh Golshan, Kristin Cadenhead, Heline Mirzakhanian

**Affiliations:** ^1^Department of Psychiatry, University of California San Diego, La Jolla, San Diego, CA, United States; ^2^Center for Integrative Medicine, University of California San Diego, La Jolla, San Diego, CA, United States

**Keywords:** first-episode psychosis, inflammation, dietary intervention, nutrition, protocol

## Abstract

**Background:**

Evidence suggests inflammation plays a role in the pathophysiology of psychosis even in early illness, indicating a potential avenue for anti-inflammatory interventions that simultaneously address high rates of metabolic disease in this population. The aim of this study is to design a novel anti-inflammatory diet intervention (DI) that is feasible to implement in a first-episode psychosis (FEP) population.

**Methods:**

Eligible FEP Participants are aged 15–30. The DI is currently being refined through a multi-phase process that includes the recruitment of focus groups that provide insight into feasibility of measures and nutritional education, as well as the implementation of the DI. The phases in the study are the Development Phase, Formative Phase, and the Feasibility Phase.

**Results:**

The Development phase has resulted in the creation of a flexible DI for FEP based on existing research on nutritional health and informed by providers. This study has just completed the Formative phase, recruiting eligible participants to join focus groups that gleaned information about dietary habits, preferences, and food environments to further refine the DI.

**Conclusion:**

Findings from earlier phases have advised the current Feasibility Phase in which this novel DI is being administered to a small cohort of FEP participants (*N* = 12) to determine acceptability of the DI from a lived experience perspective. Naturalistic changes in inflammatory biomarkers, metabolic health, and symptoms will also be measured.

## 1 Introduction

Psychosis refers to a set of symptoms that typically involves the presence of hallucinations, delusions, and profound deficits in neurocognition and functioning (1). These symptoms can occur across the lifespan and are accompanied by significant risk for chronic disability and comorbid inflammatory metabolic health conditions which will be discussed in the present section (2, 3). Importantly, individuals living with psychotic disorders such as schizophrenia or schizoaffective disorder have a 2–3 times higher mortality rate as well as a decreased life expectancy compared to healthy peers, indicating an urgent need to better understand the pathophysiology of comorbid disease and inflammation in this population and to develop appropriate interventions that address these risk factors (4, 5). The present section aims to broadly outline the current understanding of inflammation, metabolic disease, anti-inflammatory, and dietary interventions as they pertain to psychosis populations.

### 1.1 Inflammation and psychosis

Inflammation is thought to play a critical role in the development of both metabolic and psychotic disorders. Researchers have documented patterns of inflammatory cytokine dysregulation and evidence of oxidative stress in individuals with schizophrenia (2, 6–11). An inflammatory paradigm may provide a theoretical framework to inform etiological questions that surround psychosis and provide insights into targeted interventions in psychotic illness.

While studies of inflammation in psychosis are promising, findings are heterogenous across the psychosis spectrum given multiple confounders such as metabolic disturbances, chronicity of illness, substance use, and varying levels of exposure to environmental stressors (6–8, 10–13). Long-term exposure to antipsychotic medication, common in chronic psychosis cohorts, limits understanding of how inflammatory processes impact illness severity. Antipsychotics have been shown to both mediate and exacerbate inflammation, in turn contributing to the presence of metabolic syndrome in individuals with psychosis (14–18). These findings underscore the need to study the earlier stages of illness in which individuals have less exposure to these factors.

### 1.2 Need for novel interventions in early-course psychosis

Increased emphasis in recent years has been placed on studying first-episode psychosis (FEP) populations; these are individuals who have experienced a clinically significant episode of psychosis within the past 3–5 years (19). Robust clinical findings have suggested early intervention leads to improvements in symptom severity, social functioning and long-term clinical and cognitive outcomes for this population when employed within the first 5 years after the first-episode (12, 19–23). This is especially salient in the context of comorbidity of psychosis with inflammatory metabolic conditions. Individuals even in the earlier phases of psychotic illness show increased inflammatory biomarkers and higher risk for sedentary lifestyles (4, 16, 24–26). These findings suggest that psychosis could be viewed through the lens of a systemic illness in which inflammation can serve as a viable biomarker for disease risk or state. Several studies have indicated the presence of inflammatory abnormalities in both clinical high-risk (CHR) psychosis and FEP where individuals have had limited exposure to illness or medication (4, 16, 27–29).

A recent meta-analysis by Firth et al. (30) evaluated several studies (*N* = 28) measuring inflammatory characteristics of FEP populations and found that in addition to inflammatory dysregulation there is also evidence of nutritional deficiencies in FEP versus controls such as lower B vitamins (folate or B9 and cobalmin or B12), lower vitamin D, as well as some evidence of lower nutritional minerals such as zinc and magnesium. Cadenhead et al. (24) observed metabolic abnormalities among medication naive participants at CHR for psychosis as well as an association between diets low in Omega 3 and symptoms of subsyndromal psychosis. Unfortunately, current gold-standard treatment for early-course psychosis does little to consider environmental risk factors on overall health and disease progression (31). Given that the risk for severity of metabolic health issues appears to be positively associated with illness duration among individuals with FEP in particular, there is a need for anti-inflammatory intervention trials in this population that not only assess psychotic symptoms as target endpoints but as biomarkers of metabolic health (26).

### 1.3 The impact of anti-inflammatory interventions and significance for early psychosis populations

Anti-inflammatory interventions have been explored across diverse patient populations. Significant research attention has been placed on inflammation as a modifiable treatment target that can be influenced through different modalities ranging from anti-inflammatory medications to changes in one’s diet and lifestyle (3, 9, 13, 32). While the mechanisms by which inflammation impacts the trajectory of psychiatric disorders is largely unknown, there are active research initiatives to trial low-harm interventions that have demonstrated anti-inflammatory benefit and potential impact on psychiatric symptoms (3, 6, 10, 33).

The use of non-steroidal anti-inflammatory drugs (NSAIDs) to reduce systemic inflammation among chronic patients has been documented with small studies indicating NSAID intervention leads to decreased peripheral inflammatory cytokine levels and decreased psychotic symptomatology (34–38). Findings from Zhang et al. (39) demonstrated the potential value of microglia inhibitors such as minocycline in the amelioration of cognitive deficits, negative symptoms, and overall systemic inflammation among a group of individuals with chronic schizophrenia (40) (*N* = 75). Recent studies have also evaluated the anti-inflammatory effects of Omega-3, Vitamin D, mindfulness, yoga, and cannabinoids in psychosis populations, highlighting the potential clinical utility of these interventions as they are easily modifiable and they promote health and well-being (16, 24, 41, 42). Two reviews of adjunctive nutritional interventions for schizophrenia by (43, 44) outlined that there is growing evidence supporting the possible benefits of supplementation of N-acetyl cysteine (NAC), alpha lipoic acid (ALA), Melatonin, B vitamins, L-thyanine, and essential polyunsaturated fatty acids (PUFAs) on metabolic health, sleep, and in some studies, negative symptoms when used in tandem with traditional antipsychotic intervention. Joseph et al. (45) similarly suggest there is substantial preclinical and clinical evidence in schizophrenia contexts showing that the adjunctive adoption of short chain fatty acids (SCFA), which have been shown to reduce neuroinflammation, through an anti-inflammatory diet may be a viable combined intervention in schizophrenia given its emphasis on adding whole foods rather than restricting foods or food groups.

### 1.4 Dietary interventions for metabolic health

Dietary interventions (DI) that emphasize reducing inflammation through the consumption of nutritious whole foods have been investigated across medical conditions including rheumatoid arthritis, depression, and chronic schizophrenia (2, 3, 46–51). In addition to the early metabolic changes, individuals with psychosis are also more likely to have diets that are high in processed carbohydrates and refined sugars, which can imbalance the gut microbiome and increase insulin resistance (2, 3, 32). Small studies suggest dietary interventions are low-harm and have a beneficial impact on improving psychiatric symptoms as well as overall metabolic health (2, 3, 9, 47, 52). Gilbert-Jaramillo et al. (47) investigated the efficacy of a 12-week ketogenic diet for chronic psychosis and found a significant reduction in positive and negative symptoms as well as improved liver function among participants. Based on these findings, similar diets may have utility to address symptoms and health for early psychosis groups. Specifically, anti-inflammatory diets emphasize a high intake of Omega-3 fatty acids, fibrous cruciferous greens, probiotics, and a low intake of processed carbohydrates and refined sugars. These diets can regulate metabolic processes through the production of SCFA, a hallmark dysregulation in a variety of inflammatory diseases and psychiatric conditions (2, 46, 53, 54). Some dietary interventional studies also address dysbiosis (an imbalance of colonies of microbacteria in the gut), an emerging theory behind inflammatory findings in several psychiatric disorders (54–56). Dietary intervention may help to recultivate and encourage the growth of healthy gut bacteria (50, 53, 54).

A recent review by Aucoin et al. (3) evaluated 718 articles (*N* = 52,634) that explored topics such as levels of individual nutrients and vitamins common in this population, the gut microbiome, dietary macronutrients and patterns, as well as food sensitivities in various psychosis populations (3). This review developed a comprehensive summary of evidence and informed dietary recommendations for individuals with psychosis, emphasizing a reduction in processed foods and refined carbohydrates. This summary also included a list of dietary factors to increase (e.g., fruits, vegetables, high fiber foods, Omega 3 fatty acids, sources of vitamin B12, folate, vitamin B6, zinc, protein sources that are high in glycine and lysine, and vitamin C) and recommended their sources (3). Despite only involving studies of chronic psychosis populations, this novel review provided valuable psychosis-specific insight that can be adapted for early psychosis populations.

### 1.5 Proposal of a novel anti-inflammatory dietary intervention for first episode psychosis

Given socioeconomic disparities among individuals living with chronic psychotic disorders including limited access to affordable food, lack of psychosocial support to maintain dietary habits and unstable living conditions, DI are often difficult to implement and maintain overtime and its true effects are difficult to entangle from post-morbid effects associated with disease chronicity (2, 9, 32, 52, 57). The period after the FEP is considered a critical window for treatment intervention, prior to the onset of health problems, when individuals may have more access to family support networks who can assist with dietary monitoring and food preparation (22). Current recommended clinical intervention for FEP is generally limited to a combination of antipsychotics and cognitive behavioral therapy (CBT), and while these treatments are generally effective for reducing positive symptoms associated with psychosis, their impact on negative symptoms and comorbid health issues is generally negligible (58, 59). There is a need to incorporate dietary education and wellness intervention into treatment for psychosis in young people as they gain independence. Incorporation of health education is critical to the lifespan approach to identifying and treating psychosis, and there is a dearth of research aimed at studying the impact of nutritional assistance and education on clinical outcomes in this population (57, 60).

To our knowledge, only one study to date has trialed an anti-inflammatory diet among FEP specifically. Among a cohort of 33 FEP participants and aged-matched healthy controls, Vassilopoulou et al. (61) trialed adherence to a Mediterranean dietary intervention as adjunctive therapy with traditional antipsychotics. These investigators relied on self-reported intake of anti-inflammatory foods (olive oil, fermented foods) and measured baseline and endpoint metabolic markers such as blood pressure and blood glucose (61). This study found that adherence to the Mediterranean diet was lower among FEP compared to controls, however there was no difference in reported intake of fermented foods between diagnostic groups. Additionally, there were variations in blood glucose between individuals on different antipsychotic medication, warranting future study. Importantly, adherence to the Mediterranean diet was negatively associated with blood glucose among a subset of FEP who were taking olanzapine, azapine, clozapine, or risperidone specifically providing essential information related to the impact of adherence on metabolic changes in this population (61). This study provided valuable insight into the feasibility of an anti-inflammatory DI in FEP, however feasibility and acceptability were not the primary targets of this study, suggesting a need for future studies that aim to work collaboratively with patients to design a personalized dietary intervention.

Based on this background information, the present paper describes the methods to develop a DI informed by knowledge of the mechanistic and systemic biological changes observed in FEP and iterative feedback from patients and their families.

Here we describe the methods to address the following aims:

1. Design a diet intervention based on anti-inflammatory foods using continuous feedback from early psychosis patients and their families.
2. Evaluate participant adherence to/feasibility of a 5-week anti-inflammatory DI.
3. Pilot feasibility of collecting the following outcome data and biomarkers of target engagement pre/post DI:

- immune modulating (Lactobacillus and Bifidobacterium) species in gut microbial composition
- proinflammatory cytokines and lipopolysaccharide (LPS) associated inflammation
- weight and metabolic parameters
- symptoms of psychosis

This novel DI, developed through a multi-phasic small-scale feasibility study, is intended to provide a framework for future nutritional intervention trials for FEP as well as their support systems that emphasizes improvements in diet, psychiatric symptoms, general health, and is guided by lived experience and perspectives.

## 2 Methods

Participants in this study will be recruited from the University of California San Diego Cognitive Assessment and Risk Evaluation (CARE) program, an early psychosis clinic and research program. Recruited participants will complete an informed consent or assent to participate in a research study in accordance with the University of California San Diego Health Institutional Review Board (IRB). The development and implementation of this novel DI will be based on expert input from both the CARE program as well as the University of California San Diego Center for Integrative Medicine (CIM), a leading institution in nutrition research. This study will be conducted in three distinct phases following a similar process described by Bustamante et al. (46) who studied anti-inflammatory DI in rheumatoid arthritis. The first phase, the Development phase, involved active collaboration between researchers and clinicians to develop the DI. The second, or Formative, phase involves the recruitment and formation of focus groups (*N* = 25) with the aim of collecting qualitative interviews regarding FEP participants’ and their families’ perspectives on diet, nutrition, and health to refine the final DI. The third, or Feasibility, phase will pilot the DI within one group of FEP participants (*N* = 12) utilizing a pre/post design to assess the feasibility and acceptability of the DI. Sample sizes for each group were predetermined based on anticipated recruitment feasibility/availability of patients within the UCSD CARE program. In the Feasibility phase, researchers will also investigate the feasibility of collecting clinical outcome measures as well as metabolic markers such as weight and levels of biomarkers of inflammation. The following description of the proposed feasibility protocol will adhere to the Standard Protocol Items: Recommendations for Interventional Trials (SPIRIT) guidelines in which specific hypotheses and aims are stated (above), inclusion/exclusion criteria, methodology and relevant measures, location of study activities, rationale for sample size, timeline of the proposed study, as well as data management and statistical analyses (62). Per the National Institutes of Health Final Rule regarding clinical trial registration (FDAAA 801), the proposed feasibility study protocol does not meet the criteria for an applicable clinical trial (ACT) and was not pre-registered (63).

### 2.1 Inclusion, exclusion and clinical assessment

The study will include participants aged 15–30 and their support figures (e.g., family and/or partners). Eligible participants will have received a diagnosis of either a schizophrenia-spectrum disorder or an affective psychotic disorder within the past 5 years as determined by the Structured Clinical Interview for DSM-5 Disorders (SCID-5) (64). All clinical screening assessments will be administered by trained diagnostic raters with master or doctoral level degrees in clinical psychology within the UCSD CARE program. Both affective and non-affective psychoses will be included given the heterogeneity of FEP diagnoses, and that a wide range of psychosis spectrum conditions are reflective of the diagnostic makeup of the UCSD CARE program (65, 66). Full inclusion and exclusion criteria are outlined in Table 1. Importantly, individuals will remain on their current prescribed psychiatric medication as the proposed project is testing the feasibility of an adjunctive therapy. If an individual is required to initiate antibiotics at any period during study participation in the Feasibility phase (discussed below), they will continue with the DI protocol if medically indicated and antibiotic use will be documented. Individuals currently taking antibiotic medication at study screening/ entry will not be included in the study or will be recruited once antibiotic use has ceased (see Table 1 for time-course permitted). Individuals will be representative of the greater San Diego, California community and will be reflective of diverse racial, ethnic, educational, and socioeconomic backgrounds as well as neurodivergent and sexual and gender minority statuses as is naturally observed within the UCSD CARE Program. Differences in sociodemographic factors will be evaluated following the end of this feasibility study, and any limitations or trends associated with these factors (e.g., skewed socioeconomic or educational representation) will inform future dietary interventions in this population.

**Table 1:** Inclusion and exclusion criteria.

*Inclusion criteria*
Aged 15–30
FEP diagnosis within the last 5 years of unspecified psychosis, schizophrenia, schizophreniform disorder, bipolar disorder with psychotic features or schizoaffective disorder as determined by the Structured Clinical Interview for DSM-5 (SCID-5)
*Exclusion criteria*
Diagnosis of substance-induced psychosis or psychosis due to general medical condition as determined by the SCID-5
Has significant food significant sensitivities, allergies, or follows a current dietary restriction/ regimen
Antibiotic use within the past 3 months as determined via self-report and/or medical records
Diagnosis of a major neurological condition, seizure disorder, or other significant medical illness
History of a severe head injury with loss of consciousness or traumatic brain injury per patient report
IQ < 80 or severe intellectual disability per participant report
High risk for suicide as determined through the clinical screening process

**Figure 1:**
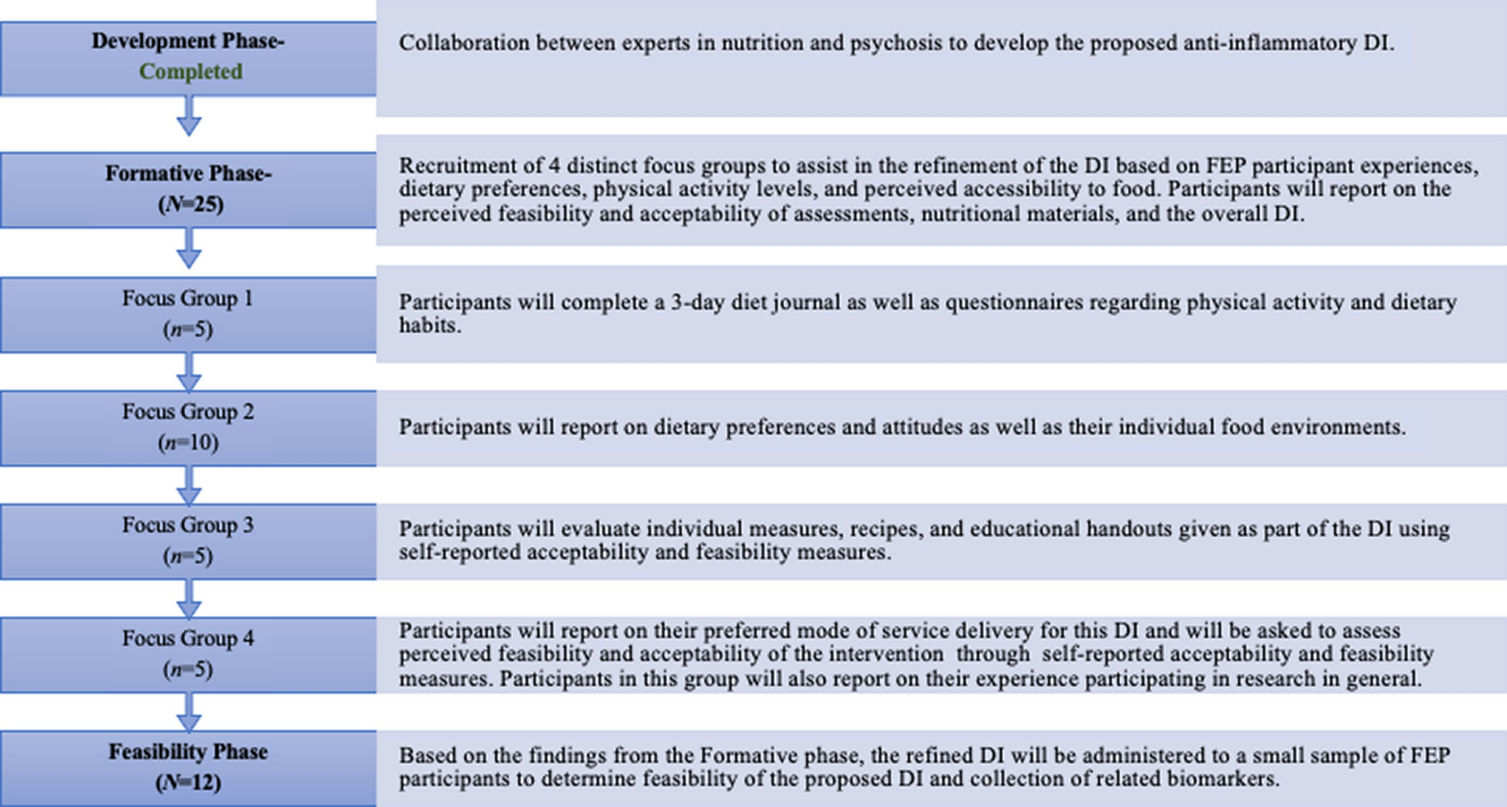
Study phases.

Individuals will be recruited chronologically for participation in distinct phases of this study which will be described in detail below (Figure 1). Severity of psychosis symptoms will be assessed at the initial screening period through the Positive and Negative Symptom Scale (PANSS), and current mood will be evaluated through the Calgary Depression Scale for Schizophrenia (CDSS) (67, 68). The Eating Attitudes Test (EAT-26) will be administered to all participants to learn more about disordered eating behavior in this population, a topic poorly understood and understudied in psychosis-spectrum disorders (69).

### 2.2 Phase I: development

In the Development phase, preliminary sample diets were dynamically created based on dietary preferences and physical needs that are specific and flexible to psychosis populations based on input from registered dieticians at the CIM as well as clinicians within the CARE clinic (Table 2). In addition to relying on research findings in this area, the delivery of this tentative DI was refined and strategized based on clinician views of perceived acceptability and feasibility of patients within the CARE clinic with whom clinicians had worked with or treated. The primary method of delivery for this DI is through remote modalities with the intention of determining whether these channels of communication are feasible and acceptable by research participants in the Formative phase of the study. During the COVID-19 pandemic, such methods of service delivery have been found to be effective in reaching diverse populations (70, 71). This method may be essential to those living with psychosis as several studies have highlighted poor in-person treatment and research adherence among FEP populations (72, 73).

**Table 2:** Sample diet plan from the UCSD CIM.

	Breakfast	Lunch	Snack	Dinner
Monday	Brain Smoothie or Strawberry Peach Kale Smoothie	Balsamic Dressing, Green Salas with Kidney Beans, Pumpkin Seeds	Guacamole and Carrot Sticks	Cauliflower-Millet Steamed Broccoli
Tuesday	Vegetable Egg Scramble	Purple Cabbage Salad	Chia Seed Pudding	Papita Pesto Nourish Bowl
Wednesday	Oatmeal	Left Over Nourish Bowl	Sweet Potato Hummus and Crackers	Baked Mahi Mahi, Roasted Vegetables, Whole Grain
Thursday	Fresh Spinach Quiche Cups	Turmeric-Ginger Dressing, Green Salad White beans	Baby Carrots and Hummus	Lentils with Vegetables
Friday	Quinoa Porridge OR Oatmeal	Left Over Lentils with Vegetables	Miso Soup	Kale Salad with Rotisserie Chicken
Saturday	Left Over Fresh Spinach Quiche Cups	Roasted Vegetables and Tahini Dressing	Plain Yogurt with Berries and Ground Flaxseeds	Steamy Greens à la Esselstyn, Left over Rotisserie Chicken, Whole Grain
Sunday	Left Over Berries with Coconut Mango Cream	Asian Coleslaw	Edamame Dip	Salmon Pecan Cakes, Roasted Brussel Sprouts

### 2.3 Phase II: formative phase

The Formative phase involved four distinct focus groups (Table 3). Each focus group in this phase provided novel information on participants’ attitudes towards health, dietary preferences, accessibility to food, feasibility, and acceptability of a DI based on lived experience. Information collected during each focus group in this phase was used to refine the DI further in preparation for the Feasibility Phase. Participants in each focus group will be evaluated remotely via telehealth within their homes and may have a family member or support figure present depending on their comfort level/preference.

**Table 3:** Study flow- formative phase.

	Assessment	Formative phase			
		Focus group 1	Focus group 2	Focus group 3	Focus group 4
General forms	Demographics	X	X	X	X
	Informed Consent	X	X	X	X
	Review of inclusion/exclusion criteria	X	X	X	X
	Medical & Family History Questionnaire	X	X	X	X
	Clinical Assessment with MD				
Clinical assessment forms	SCID	X	X	X	X
	PANSS	X	X	X	X
	CDSS	X	X	X	X
Physical evaluation tools	Anthropometrics	X	X	X	X
	Weight	X	X	X	X
	Vital Signs				
	Blood Chemistries (insulin, glucose, HgA1C, lipids, liver function test); (1ML)				
	Biomarkers of Inflammation (20ML)				
	Diet Counseling				
Dietary assessment tools	EAT-26	X	X	X	X
	ASA-24	X		X	X
	FFQ	X		X	X
	Appetite and Eating Questionnaire Motivation for Change	X	X	X	X
	Diet Habits Survey	X		X	X
	NEMS-P		X	X	X
	Cooking videos			X	X
	Diet Education materials			X	X
Physical activity assessment form	IPAQ Long	X		X	X
	Health Coaching Telephone				
Health coaching forms	Diet Adherence (HC note)				
	Concomitant medications				
	Assessment of AEs-Tolerability				
End of treatment surveys	Acceptability			X	X
	Feasibility			X	X
	Satisfaction/Perception			X	X

#### 2.3.1 Focus group I

The aim of this focus group (*n* = 5) was to collect information on past and present dietary habits as well as physical activity and exercise routines. The participants in this focus group were asked to complete a diet journal through the Automated Self-Administered 24-Hour (ASA-24) dietary recall system (74). Participants also completed the Food Frequency Questionnaire (FFQ) to elicit information on how often they consumed different categories of food (75). Current physical activity levels and exercise habits were assessed through the International Physical Activity Questionnaire (IPAQ) (USDA).

#### 2.3.2 Focus group II

The purpose of the second focus group (*n* = 10) was to illicit information on participants’ gastronomic preferences and sensitivities, knowledge of nutrition and various ingredients, and motivation to change their current dietary routine. Importantly, this focus group helped to highlight potential challenges in adherence to a dietary structure that may be unique to an early psychosis population. Participants in this group also provided input on their food environments and perceived notions of food availability and affordability within their communities. This food environment information was gleaned through the Appetite and Eating Questionnaires (AEQ) which encompassed several self-report scales: The Short Nutritional Assessment Questionnaire (SNAQ) assessed potential levels of malnutrition (76), the Food Craving Index (FCI) assessed daily cravings for specific foods (77). Additionally, this focus group was asked questions regarding emotional responses to gaining weight, eating certain types of food, and eating customs through the Three-Factor Eating Questionnaire (TEEQ) (78).

Information collected from this focus group also provided insight into an ambiguous and scarcely researched element of life for those living with psychosis: their food environments and the perceived accessibility and affordability of healthy foods in their communities. While previous findings have suggested that individuals living with serious mental illness are particularly vulnerable to food deserts and lack of access to nutritional foods, there is a dearth of culturally competent studies which have aimed to understand one’s perceived food environment, especially for those in the early phases of illness (79). The Nutrition Environment Measures Survey-Perceived (NEMS-P) was utilized to collect information on perceived nutrition environments of participants and assessed community nutrition environment, individual nutrition environment, home food environment, demographics, and shopping behaviors (80).

#### 2.3.3 Focus group III and IV

**Figure 2:**
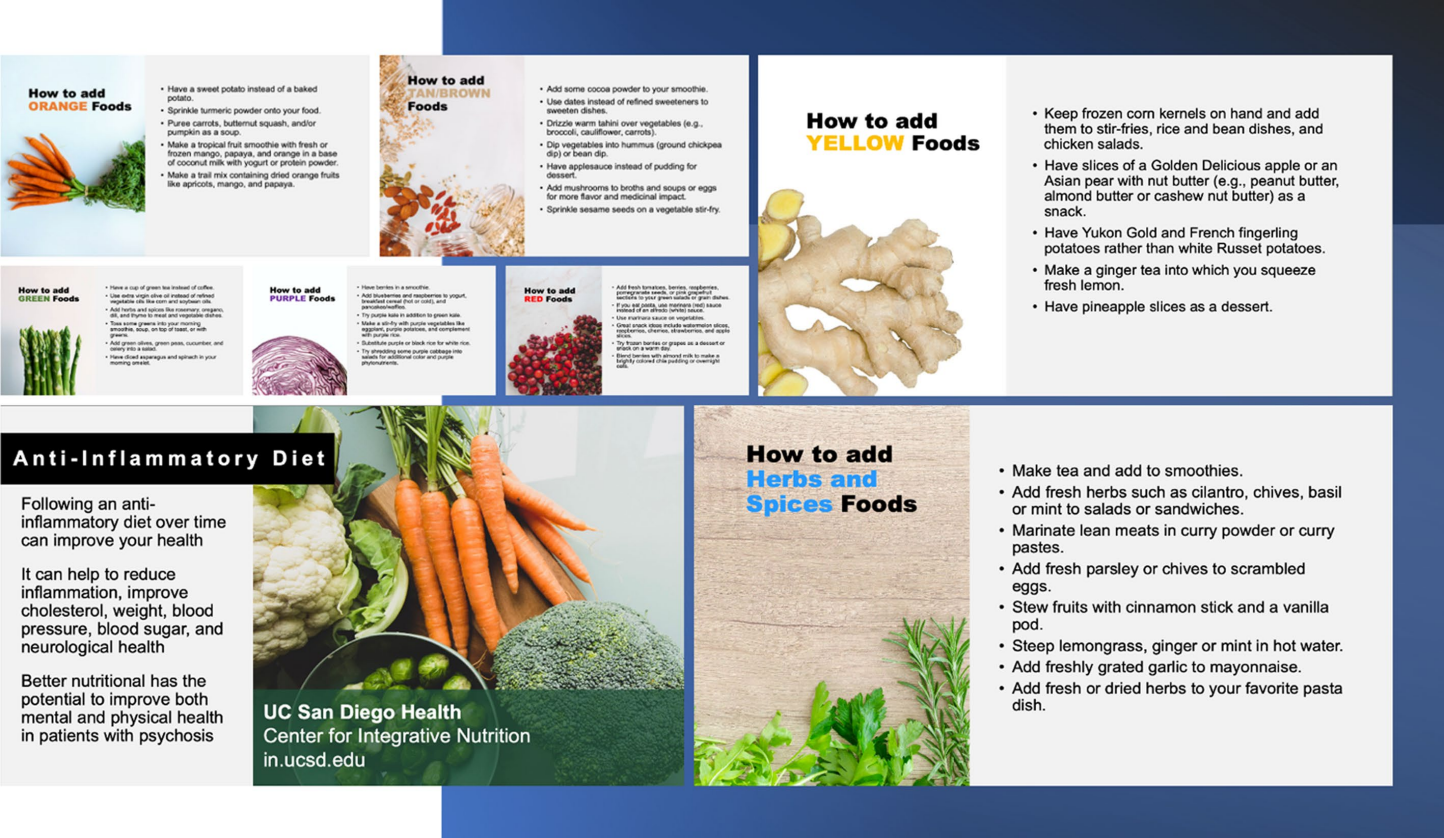
Educational handouts provided to the participant in focus groups 3 and 4.

The aim of the third focus group (*n* = 5) was to collect participant feedback on the instructional materials for the DI. This involved participants evaluating individual measures, recipes, and educational handouts given as part of the DI (Figure 2). The fourth focus group (*n* = 5) assessed overall participant satisfaction with the entire DI via the Participant Satisfaction Survey. Adapted scales from Weiner et al. (81) were used to assess the feasibility and acceptability of the intervention materials and the proposed intervention consisting of questions that required the participant to rate on a Likert scale their level of agreement with several statements regarding acceptability, feasibility, accessibility. This modified scale is included in the Supplementary material. Participants will also have the opportunity to provide unstructured qualitative insight into these aspects of the DI and research participation. Findings from these final focus groups provided insight into the readiness of the proposed DI, now further refined through an iterative process, to proceed to the Feasibility Phase. Preliminary results from the Formative phase will be reported elsewhere. Updated recipes were developed by study staff from the Center for Integrative Medicine at UCSD based on feedback from the Formative Phase. These recipes will be employed in the Feasibility Phase and are included in the Supplementary material.

### 2.4 Phase III: feasibility phase

The primary aim of the Feasibility phase is to assess the feasibility and acceptability of the 5-week anti-inflammatory DI developed in the Formative phase among a small FEP cohort (81). A secondary aim is to naturalistically evaluate the impact of the 5-week DI on weight and various other metabolic markers of inflammation as well as on clinical symptoms. The DI’s duration of 5-weeks was both informed by experience within the CIM regarding an appropriate duration to see metabolic changes, as well as input from patients in the Formative Phase of an appropriate timeline for their availability and interest. Meta-analytic findings also demonstrate that short-term dietary interventions (longer than 2 weeks) have been shown to impact gut microbiota (82), and similar reviews have noted that anti-inflammatory dietary interventions longer than 4 weeks may have observable effects on blood-based inflammatory markers (83, 84) A projected sample size of 12 for this phase was proposed *a priori* based on similar feasibility studies within the CIM, and parameters that would be measured in the Feasibility phase were selected based on responses from participants in the Formative phase. Following the Formative phase, study staff from both the UCSD CARE Program and the CIM discussed participants’ qualitative and quantitative feasibility and acceptability ratings for all assessments administered as well as educational materials provided. Study staff then discussed and decided, based on these responses, which assessments and educational materials would be administered in the Feasibility phase and reduced assessments in which there was significant overlap of content or which participants had expressed difficulty completing or understanding (Table 4). Given that the objectives of the proposed study are to assess the feasibility and acceptability of the DI as well as to explore any impact on metabolic outcomes following the DI within an FEP population, healthy control participants will not be included in this pilot study as there is no methodological question proposed in this project regarding applicability or comparability to a healthy control group.

**Table 4:** Study flow- feasibility phase.

	Assessment	Feasibility phase					
		Baseline (Week 0)	Phone-check-in (Week 1)	Phone-check-in (Week 2)	Clinical evaluation (Week 3)	Phone-check-in (Week 4)	Endpoint (Week 5)
General forms	Demographics	X					
	Informed Consent	X					
	Review of inclusion/exclusion criteria	X					
	Medical & Family History Questionnaire	X					
	Clinical Assessment with MD	X					
Clinical assessment forms	SCID-5	X					X
	PANSS	X					X
	CDSS	X					X
Physical evaluation tools	Anthropometrics	X					X
	Weight	X					X
	Vital Signs	X					X
	Blood Chemistries (insulin, glucose, HgA1C, lipids, liver function test); (1ML)	X					X
	Biomarkers of Inflammation (20ML)	X					X
	Diet Counseling	X	X				
Dietary assessment tools	EAT-26	X					
	ASA-24	X	X		X		X
	FFQ	X					X
	Appetite and Eating Questionnaire Motivation for Change	X					X
	Diet Habits Survey						
	NEMS-P	X					
	Cooking videos		X	X	X	X	
	Diet Education materials		X	X	X	X	
Physical activity assessment form	IPAQ Long	X			X		X
	Health Coaching Telephone	X	X	X	X	X	X
Health coaching forms	Diet Adherence (HC note)		X	X	X	X	X
	Concomitant medications	X	X	X	X	X	X
	Assessment of AEs-Tolerability		X	X	X	X	X
End of treatment SURVEYS	Acceptability						X
	Feasibility						X
	Satisfaction/Perception						X

Given that participants are going to be recruited directly from the UCSD CARE Early Psychosis program, we anticipate retention across the 5-week DI as study staff have regular communication and interaction with patients in the clinic and research setting. If participants withdraw or leave the study at any point during the 5-week DI, this missing data will be incorporated into our holistic understanding of the feasibility and acceptability of the DI. Participants in this study are not randomized and primarily qualitative data is being collected over the course of the DI, therefore typical methods for managing attrition issues and missing data used in clinical trials such as intention to treat (ITT) or last observation carried forward (LOCF) analyses are not necessary. FEP participants recruited to this phase will importantly be different than those who participated in the Formative phase to ensure objective exposure to final DI material. Possible barriers to attrition are also addressed through the modality in which the DI will be delivered; participants will only come into the research center physically for the baseline and endpoint collection of bio-samples, and all clinical assessment appointments and weekly diet-coaching sessions will be held remotely.

### 2.5 Data analysis, monitoring, and management

For the clinical and metabolic measures collected in the Feasibility phase (discussed below), a series of exploratory analyses will be used to estimate effect sizes and directionality of changes from pre to post 5-week DI. If basic statistical assumptions are met, the preferred method of analysis will be a one sample paired t-test between baseline and end point measures. If the final data does not meet these assumptions, an alternative Wilcoxon signed-rank will be considered. Pearson’s correlation will be used to analyze the association of average adherence to secondary outcome measures, however if the data appears to be non-parametric, alternative Spearman’s correlations will be considered. For the analysis of feasibility, acceptability and participant satisfaction of the DI, descriptive statistics will be obtained for all variables and the appropriate statistical test will be employed to assess normality and homogeneity of variance.

Per the UCSD Health IRB guidelines, participant data will be collected under de-identified subject IDs. All data will be collected and simultaneously input into a secure REDCap database formulated specifically for this study (87). Clinical assessments, demographic data, and diet coaching materials will all be stored on this secure database. Study staff will have all completed the required Collaborative Institutional Training Initiative (CITI) certification for research in Human Subjects (CITI).

## 3 Anticipated results and outcomes

### 3.1 Adherence to a 5-week anti-inflammatory DI: clinical and metabolic outcomes

Height, weight, and other markers of obesity and metabolic health will be collected pre and post DI (Table 4). Plasma lipids, fasting glucose, hemoglobin A1C (HgA1C), C-Reactive Protein (CRP), and insulin levels will be collected via blood draw at baseline and week 5. The gut microbiome for each participant will be examined pre and post intervention through the collection of fecal samples at baseline and at endpoint. Based on preexisting fecal sample collection protocols from Sinha et al. (85), samples will be obtained using Fecal Occult Blood Tests (FOBT). Levels of immune-modulating gut species *Lactobacillis* and *Bifidobactirium* will be analyzed using Shotgun metagenomics sequencing (85, 86). During this initial visit, participants will be introduced to study staff and provided with instructional material on the DI as well as a 5–7 day meal plan that will be adjusted with each week of the DI. They will be provided with dried goods that are essential for the DI, and will be given instructions on how to complete questionnaires and will gain access to online instructional cooking material.

After baseline, participants will meet once per week for 5 weeks with study staff and complete questionnaires. During these weekly check-ins, participants will meet with the registered dietician and review weekly adherence to the DI, as well as barriers and facilitators related to adherence. The registered dietician will consult on modifications to the DI as well as review sample recipes with the participant and answer any questions (Supplementary material). Medication adherence and overall health and well-being will be assessed at these visits, and participants will be encouraged to explore the available online resources of cooking videos and recipes to assist them with the DI. After the 5-week DI, participants will complete final biomarker testing and clinical evaluation.

### 3.2 Feasibility, acceptability, and adherence

One aim of the Feasibility phase of this DI will be to assess patterns of participant recruitment and retention, participant satisfaction to the DI, and to evaluate the overall feasibility of a 5-week anti-inflammatory DI among FEP cohorts. Adherence to the overall research process will be evaluated based on the number of participants that complete all timepoints of the study. Adherence to the DI will incorporate this measure as well as qualitative weekly adherence to the DI as recorded by the registered dieticians in the weekly coaching sessions. At the end of the 5-week DI, feasibility of the DI will be informed by participant self-report (81, 89). Subjects will also be asked to complete the Research Participant’s Perception Survey-UP to provide feedback into their overall experiences as a research participant in this study (89). This phase will also elucidate the feasibility of collecting and monitoring naturalistic changes in certain biomarkers that can be used to measure metabolic health in early psychosis populations, which is an area of significant interest in the study of inflammation across psychiatric disorders. Similarly, psychiatric symptoms will be evaluated pre-and-post DI as an exploratory aim. Any changes in symptoms observed following adherence to the DI will inform the relevance of monitoring psychiatric symptoms in this context.

## 4 Discussion

To date, this is the first feasibility study that aims to outline the development and design of a novel anti-inflammatory DI specifically for FEP cohorts informed by patient perspectives and preferences. Conducting targeted focus groups, researchers in this study have learned about participant histories, dietary patterns, food accessibility and affordability, and their motivation to change current health behaviors. Qualitative insights into these domains based on FEP participant feedback have begun to shed light on nutritional environments for young people living with serious mental illness. This feedback allowed for a strategic refinement of the DI, both the recipes as well as the methods of service delivery, to best suit participants in the Feasibility phase. Findings from this pilot study regarding patient attitudes towards the acceptability and feasibility of anti-inflammatory dietary interventions have the potential to provide incentive for policy change (e.g., increased funding towards the integration of nutritional services and psychiatric care) and innovations in health care delivery for individuals in the early phases of psychosis. Additionally, participants in this study will provide qualitative information that will inform potential barriers to implementation of this DI in both a research and clinical context.

### 4.1 Limitations

The proposed study has several potential limitations. Generalizability of findings from this study is limited by the singular site in which it was conducted, and findings are only representative of the participants who completed this DI and are not broadly applicable to all FEP contexts. Future clinical studies that may implement this DI, if feasibility and acceptability are determined in the proposed study, can include direct comparisons between FEP control groups receiving standard of care treatment to those receiving anti-inflammatory DI adjunctive to standard of care. These studies should ensure representation from a diverse range of FEP individuals, larger sample sizes for statistical power, as well as inclusion of multiple clinical sites from varying geographic regions. Future studies may also explore longitudinal differences in response to this DI; the duration of the proposed DI was determined *a priori* based on expertise among research staff, patient perspectives, and existing literature to be reflective of a relatively limited time-window. Findings regarding the feasibility of this adherence to this short-term DI do not speak to the long term sustainability of this intervention in this population. Importantly however, findings regarding the “appropriate” or “recommended” length or sustainability of anti-inflammatory dietary interventions are mixed (46, 49, 52).

Importantly, while evidence of the role of inflammation in the pathophysiology of psychosis is promising and highlights important biological processes associated with psychosis, it is critical to underscore that this research is in its infancy especially in the context of early-course psychosis. The aim of this study is solely to provide the preliminary blueprint of a DI for early psychosis that is formed and refined using data and feedback collected from participants, with the Feasibility phase assessing the feasibility and acceptability of the DI along with metabolic and inflammatory biomarker assessments as target endpoints. Changes in metabolic and psychiatric health in the Feasibility phase may provide insight into the potential clinical treatment model, however future clinical trials would be required to evaluate its utility beyond the present sample.

## 5 Conclusion

This study aimed to outline the methodology for designing and determining feasibility of an anti-inflammatory DI for individuals in the early phase of psychosis. Findings from this feasibility study may aid in refining the field’s understanding of whether DI are valuable as adjunctive interventions that are tolerated and accessible to early psychosis populations. Through observing clinical symptoms and naturalistic changes in inflammatory biomarkers among a small subset of FEP individuals who adhere to the proposed DI, this study may also identify relevant symptomatology, blood, and gut-microbia-based biomarkers that can be incorporated into future clinical trials of anti-inflammatory DI for psychosis populations.

## Data availability statement

The original contributions presented in the study are included in the article/Supplementary material, further inquiries can be directed to the corresponding author.

## Ethics statement

This study was approved and is monitored by the University of California San Diego Health Institutional Review Board (UCSD Health IRB) for Research involving human subjects (Protocol #800621). Eligible participants provided verbal and written consent at the time of screening into the study, and minor participants and their parents or legal guardians provided verbal and written assent when applicable.

## Author contributions

LK: Conceptualization, Methodology, Writing – original draft, Writing – review & editing. TH: Conceptualization, Writing – review & editing. AH: Conceptualization, Writing – review & editing. SG: Conceptualization, Writing – review & editing. KC: Conceptualization, Methodology, Supervision, Writing – review & editing. HM: Conceptualization, Investigation, Methodology, Supervision, Writing – original draft, Writing – review & editing.
