# Supplementary material for: Development of an anti-inflammatory diet for first-episode psychosis (FEP): a feasibility study protocol
Source: Front Nutr. 2024 Jul 26;11:1397544. doi: 10.3389/fnut.2024.1397544 (PMC11310932; doi:10.3389/fnut.2024.1397544)
Supplement: Supplementary file 1 [file Data_Sheet_1.PDF]

Supplementary Material; Kennedy et al., 2024:

1. Modified Feasibility and Acceptability
2. Sample Recipes

Acceptability, Appropriateness and Feasibility of the Anti-Inflammatory Wholesome Foods Dietary Intervention (DI) Scales

Response Scale:
1 = Completely disagree, 2 = Disagree, 3 = Neither agree nor disagree, 4 = Agree, 5 = Completely agree

Acceptability of Intervention Measure (AIM)
1) The DI meets my approval.
2) The DI is appealing to me.
3) I like the DI
4) I welcome the DI.

Intervention Appropriateness Measure (IAM)
1) The DI seems fitting.
2) The DI seems suitable.
3) The DI seems applicable.
4) The DI seems like a good match.

Feasibility of Intervention Measure (FIM)
1) The DI seems implementable.
2) The DI seems possible.
3) The DI seems doable.
4) The DI seems easy to use.

References
Proctor, E., Silmere, H., Raghavan, R., Hovmand, P., Aarons, G., Bunger, A., Griffey, R., & Hensley, M. (2011). Outcomes for implementation research: Conceptual distinctions, measurement challenges, and research agenda. *Administration and Policy in Mental Health and Mental Health Services Research, 38*, 65-76. doi: 10.1007/s10488-010-0319-7

Weiner, B. J., Lewis, C. C., Stanick, C., Powell, B. J., Dorsey, C. N., Clary, A. S., Boynton, M. H., & Halko, H. (2017). Psychometric assessment of three newly developed implementation outcome measures. *Implementation Science, 12*(108), 1-12. doi: 10.1186/s13012-017-0635-3

UC San Diego
CENTERS FOR INTEGRATIVE HEALTH

Anti-Inflammatory Wholesome Foods Dietary Intervention in First Episode Psychosis Study

An anti-inflammatory diet is a way of eating and cooking using whole foods. The primary foods in this diet are whole grains, vegetables, fruits, nuts, seeds, legumes, beans, fish, and herbs and spices. Other foods such as poultry, eggs, cheese, yogurt, meats and sweets should be consumed sparingly and in small portions. Drink plenty of water daily and as desired enjoy tea or coffee.

Foods to Eat

Vegetables

- Aim for 1-6 servings of vegetables spread throughout the day at either meals or snacks.
- Favor non-starchy vegetables to cover 50% of your plate at each meal (ex: leafy greens, lettuce, tomato, asparagus, zucchini, bell peppers, etc.)
- One serving is 1 cup raw or ½ cup cooked

Fruit

- Aim for 1-3 servings of fruit spread throughout the day at either meals or snacks
- One serving is ½ cup cut or a medium-sized piece of fresh or frozen fruit, or ¼ cup dried fruit

Whole Grains or Starchy Vegetables

- Choose colorful varieties of starchy vegetables or whole grains as a 25% of your plate at each meal
- Choose colorful starches such as sweet potatoes, colorful fingerling potatoes, carrots, butternut squash, beets, and peas
- Choose unprocessed whole grains such as brown rice, wild rice, quinoa, farro, and oats or whole grain products such as whole wheat pasta
- Choose products with "100% whole grains" listed first or second in the ingredient list on the food package
- One serving is ½ cup cooked grain (ex: brown rice or whole wheat pasta), 1 slice 100% whole grain bread or 1 cup uncooked cereal grain (ex: shredded wheat)

Plant Protein-rich Foods, Low-fat Animal Foods, Fish, or Seafood

- Choose rich wholesome protein sources such as lentils (green, red, black), beans (garbanzo, black, pinto, red, white, etc.), salmon, sardines, tuna, herring, shrimp and other seafood, chicken breast, turkey, and eggs
- One serving is 1/4 cup tofu or tempeh, 1 cup soy milk, 1 egg or 2 egg whites, 1/2 cup cooked dry beans or peas
- One serving is 4 ounces poultry, or 4-6 ounces fish and seafood

Healthy Fats

- Include naturally occurring fats in nuts, seeds, nut or seed butters, and nut or seed milks, olive oil, olives, and avocados
- Other flavor-rich options are homemade dressings or sauces with olive oil and vinegar
- One serving is 2 tablespoons nuts or seeds and their butters or oils, ¼ to ½ avocado

Water, Tea, Herbs and Spices

- Drink water throughout the day. For more flavorful beverages try tea (green, herbal, white, black) or coffee
- Add delicious flavors and beneficial nutrients from herbs (rosemary, thyme, basil, cilantro, etc.), spices (turmeric, clove, ginger, cinnamon), and flavor-rich culinary foods (garlic, onion, lemon, lime)

Foods to Avoid

Added Sugar

- Avoid or limit sweetened beverages including juice, soda, sports drinks, coffee drinks, and alcohol
- Consume sweets, candies, ice cream, pre-packaged desserts, and baked goods/pastries rarely
- Consider eating fruit for dessert
- Consider reading food labels to avoid foods with grams of sugar listed under "added sugar"

Full-fat Milk, Yogurt and Cheese

- Choose lower fat varieties or plant-based alternatives (almond milk or yogurt, soymilk or yogurt)
- Change up your meals by selecting foods that taste good without the addition of cheese or try a home made dressing instead
- Limit by choosing smaller portions of these foods such as toppings to a salad rather than a main ingredient

Red and Processed Meat

- Please avoid or significantly limit all red meat and processed meats such as bacon, sausage, hot dogs, bologna, pepperoni and salami
- Replace red meat with lean meats, fish and seafood, or high-protein plant foods such as beans and legumes combined with whole grains

Refined Grains

- Refined grain is grain or grain flour that has been modified to remove the bran and germ, which are the most nutritious parts of the grain
- Decrease refined grains such as white flour, white bread, white rice, and many processed food

Total Saturated Fat

- Decrease red meat, butter and stick margarine, cheese, pastries and sweets, and fried foods such as French fries or fried chicken, and fast food
- Avoid or significantly limit high fat processed foods such as potato chips, microwave popcorn, nacho cheese dip, and American cheese slices

UC San Diego
CENTERS FOR INTEGRATIVE HEALTH

Meal Plan

	Breakfast	Lunch	Snack	Dinner
Monday	Brain Smoothie or Strawberry Peach Kale Smoothie	Balsamic Dressing Green Salad with Kidney Beans, Pumpkin Seeds	Guacamole Carrot Sticks	Cauliflower-Millet Mash Steamed broccoli
Tuesday	Vegetable Egg Scramble	Purple Cabbage Salad	Chia Pudding	Papita Pesto Nourish Bowl
Wednesday	Oatmeal	LO Nourish Bowl	Sweet Potato Hummus Crackers	Baked Mahi Mahi Roasted Vegetables Whole Grain
Thursday	Fresh Spinach Quiche Cups	Turmeric-Ginger Dressing Green Salad White beans	Baby Carrots Hummus	Lentils with Vegetables
Friday	Quinoa Porridge OR Oatmeal	LO Lentils with Vegetables	Miso Soup	Kale Salad Rotisserie Chicken
Saturday	LO Fresh Spinach Quiche Cups	Roasted Vegetables Tahini Dressing	Plain Yogurt Berries Ground Flaxseeds	Steamy Greens à la Esselstyn LO Rotisserie Chicken Whole Grain
Sunday	LO Berries with Coconut Mango Cream	Asian Coleslaw	Edamame Dip	Salmon Pecan Cakes Roasted Brussels Sprouts

UC San Diego
CENTERS FOR INTEGRATIVE HEALTH

RECIPES

Brain Smoothie

Makes 1 serving (about 12 ounces)

- 1 cup blueberries
- 1/2 cup Concord or red grapes
- 1 cup fresh dark leafy greens (e.g., arugula, collard, kale, spinach, etc.)
- 2 to 3 tablespoons **extra-virgin olive oil** (varieties such as pique, coratina, koroneiki, etc.)
- 1/2 to 1 teaspoon **turmeric**, ground
- 1 medium orange, peeled (optional)

Directions

1. Put all in a blender, and mix well.

Strawberry Peach Kale Smoothie

Makes 2 servings

- 2 cups unsweetened almond, hemp, or coconut milk
- 1 cup frozen strawberries (no sugar added)
- 1 cup frozen peaches (no sugar added)
- 2 cups fresh kale or spinach
- 1 tablespoon ground flax or chia seed
- 2 scoops vanilla protein powder (whey or vegan varieties, like pea, rice, organic soy or hemp)
- 1 teaspoon vanilla extract (optional)

Directions

1. Put all in a blender, and mix well. Add ice to make smoothie slushier, if desired.

More than Just Oatmeal

Makes 1 serving

Ingredients

- 1/2 heaping cup rolled oats (55 grams)
- 1 cup frozen riced cauliflower
- 1 tablespoon chia seeds
- 1 cup unsweetened almond milk
- 1 scoops vanilla protein powder (whey or vegan varieties, like pea, rice, organic soy or hemp)
- 1/2 teaspoon cinnamon

Directions

1. Add the rolled oats, cauliflower rice, chia seeds, and milk to a small saucepan over medium-low heat and mix well to combine. Keep it on a low simmer for 5-7 minutes until oats and cauliflower rice are softened and most of the liquid is absorbed.
2. Stir in your protein powder and cinnamon. Add more liquid if needed. Cook for 1-2 more minutes until everything is combined and warmed through. Top with toppings and enjoy!

Blueberry Cauliflower Oatmeal

Ingredients

- 1.5 cups rolled oats use certified gluten free if needed
- 3/4 cup riced cauliflower, frozen add more or less based on your preference
- 1 cup milk of choice
- 1 cup water
- 2 tablespoons maple syrup or honey
- 1/2 teaspoon cinnamon
- 1 cup frozen blueberries or to taste

Directions

1. Combine oats, riced cauliflower, milk, water, maple syrup/honey, and cinnamon in a medium sauce pot. Stir and heat on medium heat until the mixture starts to simmer.
2. Cook for 5 minutes, stirring occasionally to prevent burning.
3. Add frozen blueberries and stir in to incorporate. Cook for 1-2 more minutes.
4. Serve warm topped with more blueberries, some sliced bananas, a drizzle of nut butter or more maple syrup/honey.

Quinoa Porridge

Makes 4 servings

- 1 cup dried Quinoa (any variety)
- 1 ½ cups water
- 4 chopped dried figs or dates (or ¼ cup raisins)
- ¼ teaspoon cinnamon (and/or nutmeg or cardamom)
- Topping: Walnuts or other nuts of choice; and/or sliced fruit or fruit compote
- Optional: Non-dairy milk (almond, coconut, soy, etc.)

Directions

5. Start heating your water in a medium-sized pot, while you wash your quinoa with a strainer.
6. Add quinoa and dried fruit to heating/heated water.
7. Simmer quinoa porridge for 15 minutes, until soft.
8. Serve in individual bowls. Add non-dairy milk, if desired, and top with fruit and nuts.

Fresh Berries with Coconut Mango Cream

Makes 4 servings

- ⅔ cup coconut milk (canned)
- 1⅓ cup diced frozen mango (do not defrost)
- 1 teaspoon vanilla
- 2 cups fresh blueberries or blackberries
- Garnish: 4 mint leaves (optional)

Directions

1. To a blender, add coconut milk and frozen mango. Blend on high until smooth.
2. Add vanilla and blend again for several seconds.

3. Evenly divide berries among four dishes. Top with coconut cream.
4. Garnish with a mint leaf, if desired.

Tips: For a variation, add ⅓ cup frozen raspberries to coconut milk and mango (step 1). The pink color is beautiful on top of the berries.

Vegetable Egg Scramble

Makes 1 serving

- 2 large eggs, pasture-raised if possible
- 1 tablespoon water
- 1 teaspoon extra-virgin olive oil
- 1 cup assorted chopped raw vegetables (onions, red bell peppers, tomatoes, broccoli, zucchini, summer squash, asparagus, mushrooms, etc.)
- 1 pinch sea salt
- 1 pinch freshly ground black pepper
- 2 tablespoons chunky tomato salsa

Directions

1. In a small bowl, whisk together the eggs and water until well-mixed.
2. In a small cast iron pan, heat the oil over medium heat, and add the vegetables. Saute until the vegetables are tender but still crisp (about 2–3 minutes).
3. Add the eggs by pouring over the vegetables. Cook, stirring constantly, until the eggs are scrambled and set.
4. Season with sea salt and black pepper, and top with the salsa.

Fresh Spinach Quiche Cups

Makes 6 servings (1 serving = 2 muffin quiches)

- 3 large eggs (omega-3 variety)
- 1/2 cup cottage cheese (1% fat)
- ¼ cup feta cheese
- 2 cups fresh chopped spinach
- 1/2 cup chopped red bell pepper
- 1/4 cup chopped onion
- 3–4 drops hot pepper sauce (optional)
- 1/2 teaspoon garlic powder (or 1 clove garlic, minced)
- 1 pinch sea salt
- 1 pinch black pepper

Directions

1. Line a muffin pan with foil baking cups. Spray the cups with cooking spray.
2. Whisk eggs, and mix with cottage cheese, feta, spinach, bell peppers, chopped onion, hot pepper sauce, garlic, sea salt, and pepper. Mix well.
3. Pour evenly into 12 muffin cups. Bake at 350°F for 20 minutes or until a knife inserted in the center comes out clean.

Tips: May be frozen and reheated in the microwave, if desired (remove foil muffin cup if microwaving). Also note, any combination of vegetables may be used.

Guacamole

Makes 4 servings

- 2 cloves garlic, minced (≈ 2 teaspoons)
- 3 scallions or red onion, minced (≈ 1/4 cup)
- 1/4 jalapeno, minced
- 2 avocados, peeled
- 1 tablespoon fresh lime juice (juice of 1/2 a lime)
- 2 tablespoons chopped fresh cilantro
- 1 pinch of sea salt

Directions

1. In a medium bowl, combine the garlic, scallions, and jalapenos.
2. Add avocado and mash using the back of a fork.
3. Gently stir in lime juice.
4. Finish with cilantro and sea salt.

Sweet Potato Hummus

Makes 8 servings (1 serving ≈ ⅓ cup)

- 1 large sweet potato (12–14 ounces), cooked and mashed
- 1 can (15 ounces) chick peas, drained, rinsed
- 1/4 cup tahini
- 1/4 cup fresh lemon juice
- 3 tablespoons extra-virgin olive oil
- 1 small clove garlic, halved
- 1 1/2 teaspoons fine sea salt
- 1 teaspoon ground cumin
- 1/2 teaspoon cinnamon (optional)

Directions

1. Combine all ingredients in a food processor, and puree until smooth.

Tips: Serve with vegetables, whole grain pita bread, or seed crackers. To reduce sodium content per serving, cut added sea salt to half of current amount, add some pepper, or increase the other spices to desired taste.

Kale Salad

Makes 6 servings (1 serving ≈ 1 cup)

- 1 bunch kale
- 1/2 teaspoon sea salt
- 1/4 cup diced red onion
- ⅓ cup currants, raisins, or dried cranberries or cherries
- ⅓ cup diced apple (about . an apple)
- ⅓ cup sunflower seeds, toasted
- 1/4 cup olive oil
- 2 teaspoons red wine vinegar or unfiltered apple cider vinegar

Directions

1. De-stem kale by pulling leaves away from stems. Wash leaves, spin or pat dry. Stack leaves, roll up and cut into thin ribbons. Put kale in a large mixing bowl.
2. Add salt and massage it into the kale with your hands for 2 minutes (skipping this step will leave you with tough, stringy kale).
3. Stir onions with dried fruit, apple, and sunflower seeds into the kale. Dress with oil and vinegar.
4. Taste for sea salt and vinegar, adding more if necessary. Also taste a few bites to see if balance of sweet/sour/crunchy/chewy are all well mixed. Add extra of what you miss.

Tips: Add some baked, grilled, or rotisserie chicken to a double portion of salad for a nice meal.

Purple Cabbage Salad

Makes 6 servings (1 serving ≈ 1 cup)

- 1 small head of purple cabbage
- 3 carrots, shredded
- 1 tablespoon balsamic vinegar
- 1 1/2 tablespoons unseasoned rice vinegar
- 1 tablespoon water
- 1/4 teaspoon sea salt
- 1/4 teaspoon pepper
- 1 tablespoon olive oil
- 1/4 cup slivered almonds
- 1 whole ripe avocado, cut into chunks (optional)
- 1 cup pink grapefruit sections, cut in half

Directions

1. Core the cabbage, and process through the slicing disc of a food processor (or slice thinly to make strips). Shred carrots by hand or food processor. In a large bowl, toss together cabbage and carrots.
2. In a small bowl, whisk together both vinegars, water, sea salt and pepper. Slowly drizzle the oil in while whisking to emulsify. Pour over cabbage and carrots, and toss. Allow dressing to marinate salad for 30–60 minutes before serving.
3. Just before serving, toss the cabbage mixture with the almonds, grapefruit sections and fresh avocado.

Steamy Greens

Makes 1 serving

- 2 cups green leafy vegetables (such as kale, collard greens, beet, mustard and carrot greens, cilantro, parsley, spinach, Swiss chard, arugula, cabbage, Brussels sprouts, broccoli, cauliflower and asparagus), cut into bite-sized pieces
- 1 cup spring water
- Dash of sea salt (optional)
- Squeeze of lemon juice
- Drizzle of favorite vinegar (balsamic, brown rice vinegar, etc.)

Directions

1. Fill a medium size saucepan or steamer basket with insert into saucepan.
2. Heat up water until simmering and place greens in pan. Sprinkle a dash of sea salt (optional). Steam until bright green in color.
3. Season the greens with a few drops of lemon juice and your favorite vinegar. Enjoy often.

Roasted Brussels Sprouts

Makes 4 servings

- 4 cups Brussels sprouts, cleaned and halved or quartered
- 2 cloves garlic, minced (about 2 teaspoons minced)
- 1 small apple, peeled, cored and cut into eighths
- 1 tablespoon extra-virgin olive oil
- 1/4 teaspoon sea salt
- 1/4 teaspoon black pepper

Directions

1. Preheat oven to 375° F.
2. In a large bowl, toss together all ingredients.
3. Pour out into a cookie sheet lined with parchment paper, and spread mixture evenly in a single layer.
4. Roast uncovered for 20 minutes.

Cauliflower-Millet Mash

Makes 6-8 servings

- 4 cups or 1 medium head of cauliflower
- 1 cup hulled millet (washed and drained)
- 4 cups of water (or vegetable broth)
- 1 teaspoon cold pressed extra virgin olive oil or sesame oil
- 1 teaspoon garlic powder
- ½ teaspoon sea salt
- ½ cup parsley for garnish

Directions

1. Sauté millet, garlic powder, and sea salt in a little olive oil or sesame oil on medium-high flame, until water is fully evaporated and millet starts to toast.
2. Add in water or vegetable stock, and herbs, if using, and bring to the boil. Stir in chopped cauliflower and then simmer for about 40 minutes, or until cauliflower and millet is cooked through.

3. You may serve chunky as-is, or mash with a potato masher or a stick blender.
4. Stir in finely chopped parsley for a bit of color before serving.

Tips: Top with your favorite gravy (see "mushroom gravy" recipe) or sprinkle with *gomashio* (sesame seed + seaweed spice blend).

Salmon Pecan Cakes

Makes 8 servings (1 serving = 1 salmon patty)

- 1 3/4 cups pecans
- 1 can (7.5 ounces) wild salmon, drained
- 2 eggs
- 3 small scallions, chopped
- 1 small celery stalk, chopped
- 1 tablespoon extra-virgin olive oil
- 1 tablespoon lime juice
- 1/2 teaspoon sea salt
- 1 pinch paprika

Directions

1. Preheat oven to 350° F.
2. In a food processor, grind pecans to a fine texture.
3. Add remaining ingredients to food processor, and pulse to combine.
4. Remove mixture from food processor, and separate into eight medium patties. Place on a lightly oiled baking tray, and bake until golden, about 25–30 minutes.

Turmeric-Ginger Dressing

Ingredients

- 3 inches fresh turmeric root or 1 tablespoon turmeric powder
- 1 inch fresh ginger
- 2 cloves garlic
- 4 tablespoons raw honey
- ½ cup raw apple cider vinegar
- 2 tablespoons organic extra virgin olive oil
- 1 teaspoon mustard seeds
- 2 sprigs of scallions or green onions
- Garnish with 2 sprigs fresh basil leaves
- Optional: 3 dashes of black pepper and/or 1 dash of cayenne pepper

Directions

1. Blend all ingredients (except if using Basil) in blender or food processor, until smooth.
2. Garnish with thin slices of fresh basil leaves, if using.

Creamy Kale with Pepita Pesto

- ½ cup peptias

- 2 small garlic cloves
- 1 packed cup chopped kale
- 1 packed cup cilantro
- ¼ cup lemon juice
- ½ teaspoon sea salt
- Fresh ground black pepper
- ½ cup extra-virgin olive oil
- ½ cup water
- ½ teaspoon maple syrup

Directions

1. Blend all ingredients in blender or food processor, until smooth.

Build in bowl with ½ cup of intact kernel gain of choice (rice, millet, quinoa), green vegetables (arugula, green beans, asparagus, peas)

Chia Seed Pudding

- 6 Tablespoons chia seeds
- 1 cup coconut milk
- 1 cup water or almond milk
- ½ teaspoon vanilla extract
- ½ cup sliced strawberries
- ½ cup raspberries
- ½ cup blueberries
- ¼ cups dark chocolate chips (optional)

Directions

1. In a bowl, mix together chia seeds, coconut milk, almond milk and vanilla.
2. Once the chia pudding mixture is well combined, let it sit for 5 minutes, give it another stir/whip to break up any clumps of chia seeds, cover and put the mixture in the fridge to “set-up" for 1-2 hours.
3. Wash strawberries, raspberries, blueberries set aside
4. To serve alternate layers of pudding and berries in a cup or small bowl

Tahini- Garlic Sauce

- 1 container of silken tofu
- ¼ cup chickpea miso
- 1/8 teaspoon toasted sesame oil
- 1 teaspoon Tahini (sesame seed butter/paste)
- 1 teaspoon dehydrated garlic or garlic powder
- ¼ cup hot, boiling water

Optional addtions:

- dill and lemon (1/4 lemon, squeezed juice)
- grated ginger and toasted sesame seeds
- green onion, chopped fine

- cilantro, parsley, and a few dashes of cumin

Directions

1. Blend all ingredients until smooth.
2. Drizzle on top of favorite veggies or brown rice.

Miso Soup

- 4 cups vegetable broth or spring water
- 1 inch piece of Wakame sea vegetable cut into a 1-inch square pieces
- ¼ cup of onion chopped fine (or other round vegetable)
- 1 dried shiitake mushroom soaked in spring water and chopped finely
- ½ cup sliced daikon radish
- 1/8 pound firm tofu, cut in 1/4-inch cubes
- ½ carrot julienned (or other root veg)
- 1 tsp barley miso (brown in color)
- 1 green onion/scallion/chives or parsley, chopped fine, for garnish

Optional:
1 broccoli floret or other piece of green (collards, kale, bok choy)
¼ cup cubed mochi

Directions

1. Put water in a saucepan with sea vegetable. Bring broth/water to a boil, add chopped onion and simmer until clear. Add mushrooms, carrot, daikon, and tofu cubes. Simmer until soft. Add greens at the end of cooking.
2. Transfer about 1 cup broth from the pot into a measuring cup and add miso. Stir with a spoon until completely dissolved, then return mixture to pot. Stir to mix, garnish and serve.

Baked Mahi Mahi

Ingredients (2 portions)

- Mahi-mahi (3 1-2 inch steaks per person). (If it is frozen, remove it from the freezer the night before and put it in the refrigerator to thaw.)
- Turmeric
- Black pepper
- Half of a lemon (for squeezing)

Directions

1. Preheat the oven to 425F.
2. Line a baking sheet with parchment paper or silicone mat.
3. Pat dry the fillets and sprinkle with the spices and a pinch of salt on both sides.
4. Cook the fish for 15 minutes. Remove from oven and squeeze the lemon juice over the fish. Serve hot.

Asian Coleslaw

- ½ head of cabbage (red, green, Chinese, or savoy)
- 1 large carrot, shredded
- Lemon juice (use 1 lemon = 3 tablespoons)
- 2 tablespoons miso
- ¼ cup water
- 2 tablespoons brown rice vinegar
- 3 tablespoons toasted sesame seeds (hand-toast right before tossing for maximum flavor)
- Optional: dash of Ume boshi plum vinegar

Directions

1. Chop cabbage into fine slivers, and shred or julienne carrots.
2. In a small bowl, dissolve miso in vinegar and lemon juice and/or umeboshi plum vinegar.
3. Toss sauce with cabbage and carrots.
4. Sprinkle with toasted sesame seeds.

Lentils with Vegetables

Cook time: 2 hours

Ingredients (2 portions)

- 7 ounces lentils, uncooked
- 1 medium sweet potato (optional)
- 2 large carrots
- 1 onion
- 1 clove of garlic
- 1 bay leaf
- 1 teaspoon of salt
- 1 pinch of black pepper
- 1 pinch of turmeric
- ½ leek
- Water
- ½ green bell pepper
- 1 tablespoon of virgin olive oil

Directions

1. The night before, wash the lentils by soaking in cold water (cover the lentils with twice the volume of water). If you forget to do this step, wash them the same way and add 30 minutes to the cooking time.
2. Peel and chop all of the vegetables into small pieces.
3. In a large casserole dish, put two spoonfuls of virgin olive oil, the onions, the leek and the clove of garlic. Fry on low heat for 3 minutes.
4. Add the pepper, the sliced carrot and the bay leaf and continue to cook on low heat for two minutes more.
5. Add the spices and stir with a wooden spoon so that it does not burn. Let the lentils drain and mix.
6. Add cold water to generously cover the lentils (about 3-4 cm above the lentils). Allow it to sit on the heat for 30 minutes on medium heat. Then add the salt and cook for another 30 minutes.
7. Bring it to a simmer for a final 30 minutes.

Balsamic Vinaigrette

Makes ¾ cup

- ¼ cup balsamic vinegar
- ½ cup extra-virgin olive oil
- ¼ teaspoon salt

Directions:

1. Combine all ingredients in a jar with a tight-fitting lid and shake until well-blended, or whisk ingredients in a bowl.

Edamame Dip

- 12 oz cooked and cooled edamame
- ½ cup chopped green onion
- 1 clove garlic
- ½ cup parsley
- 1 tbsp miso paste
- 1 tsp red chili paste
- ¼ cup lemon juice
- 1 tsp salt
- 5 tbsp olive oil

Directions

1. Blend all ingredients in blender or food processor, until smooth.
